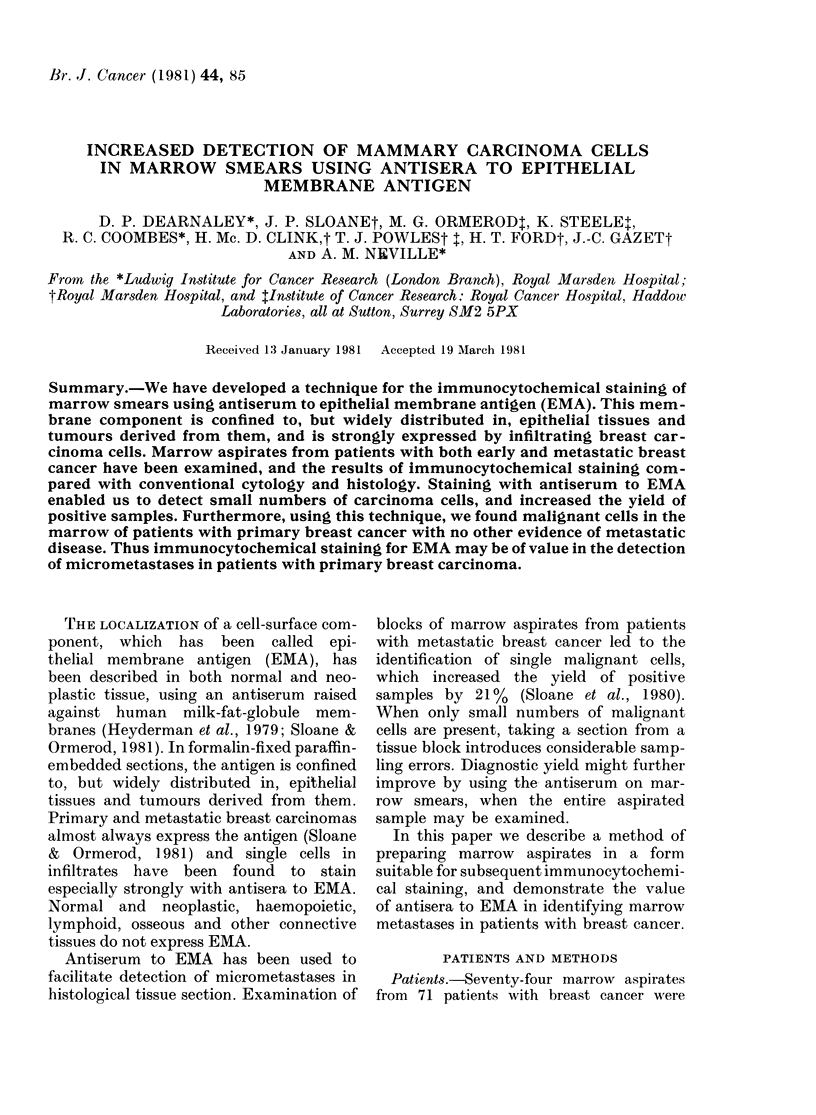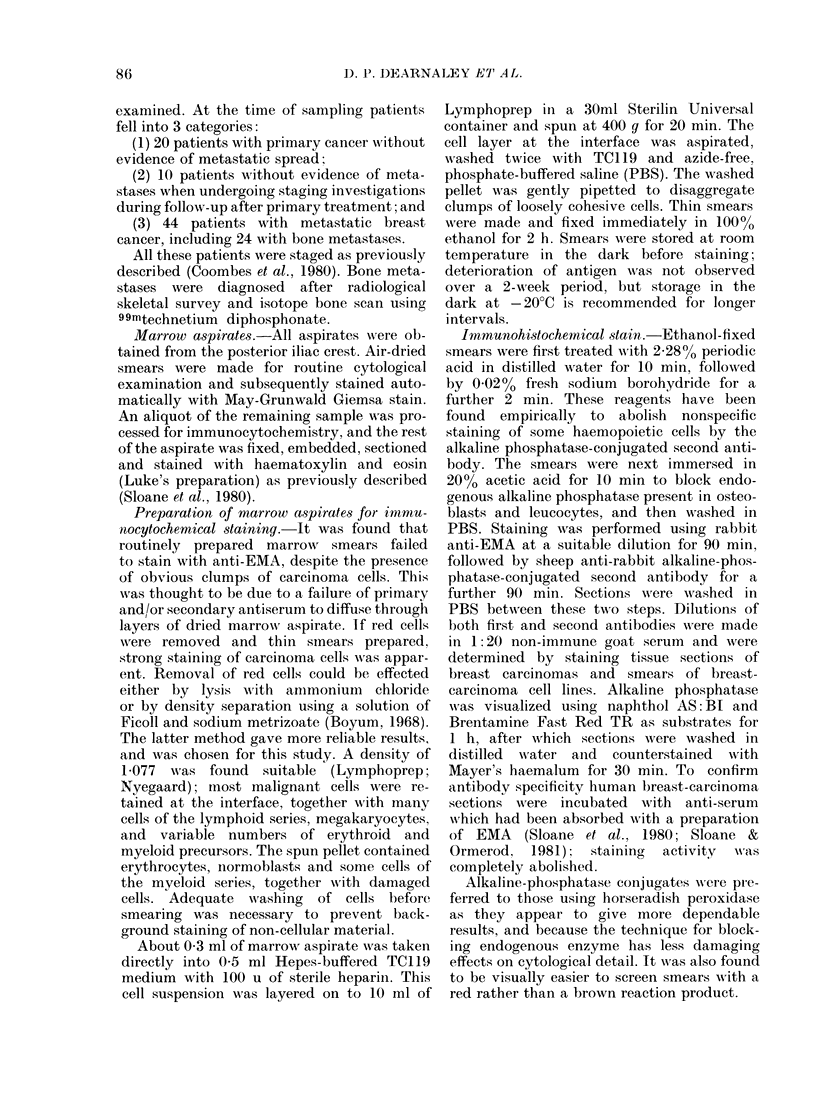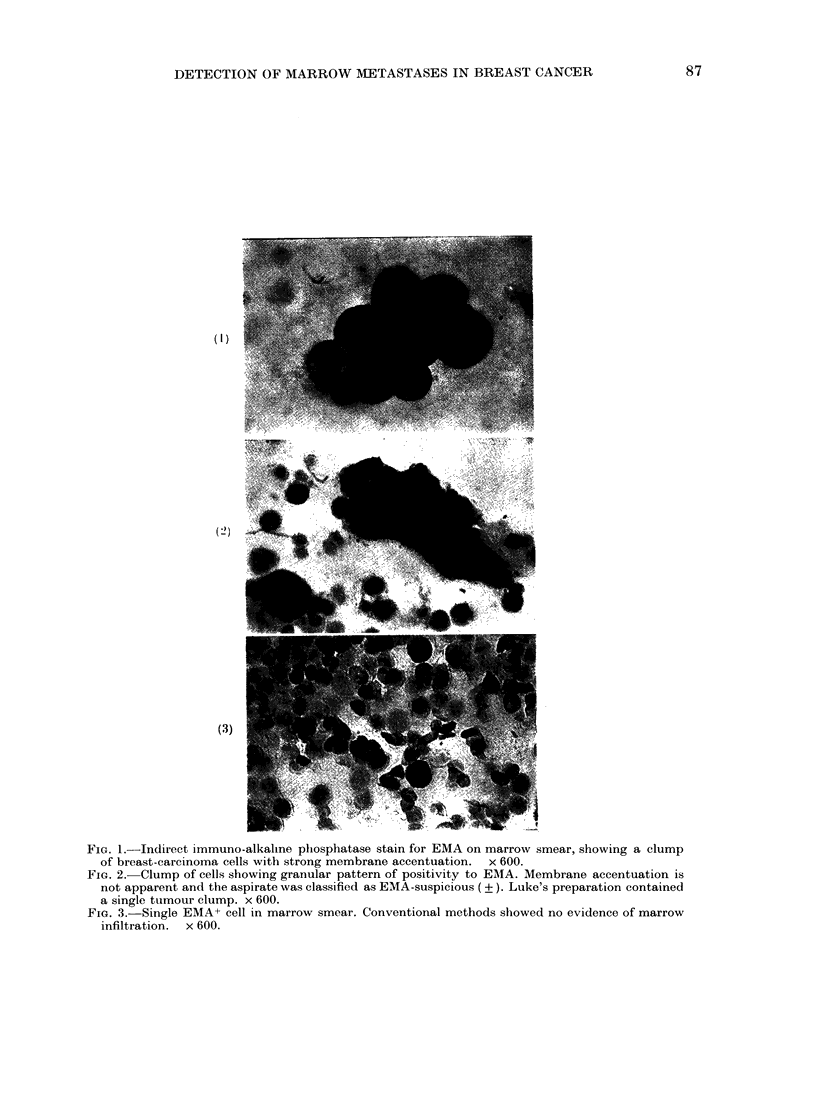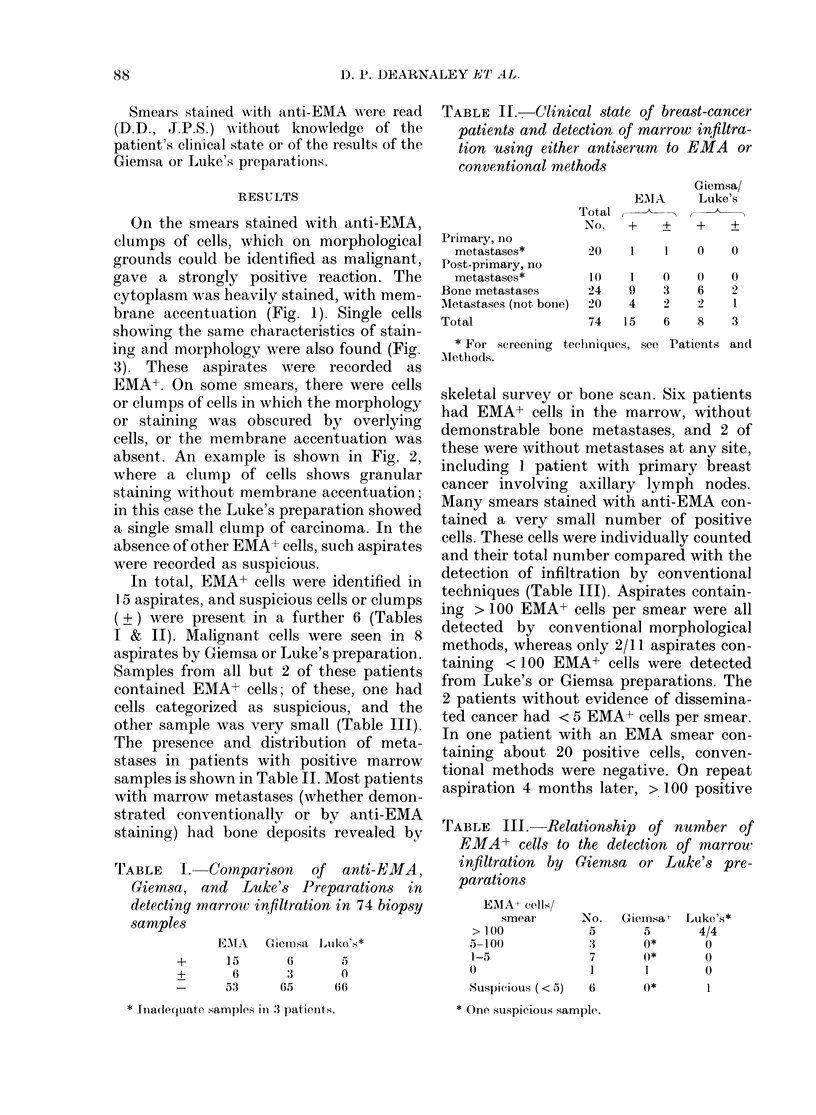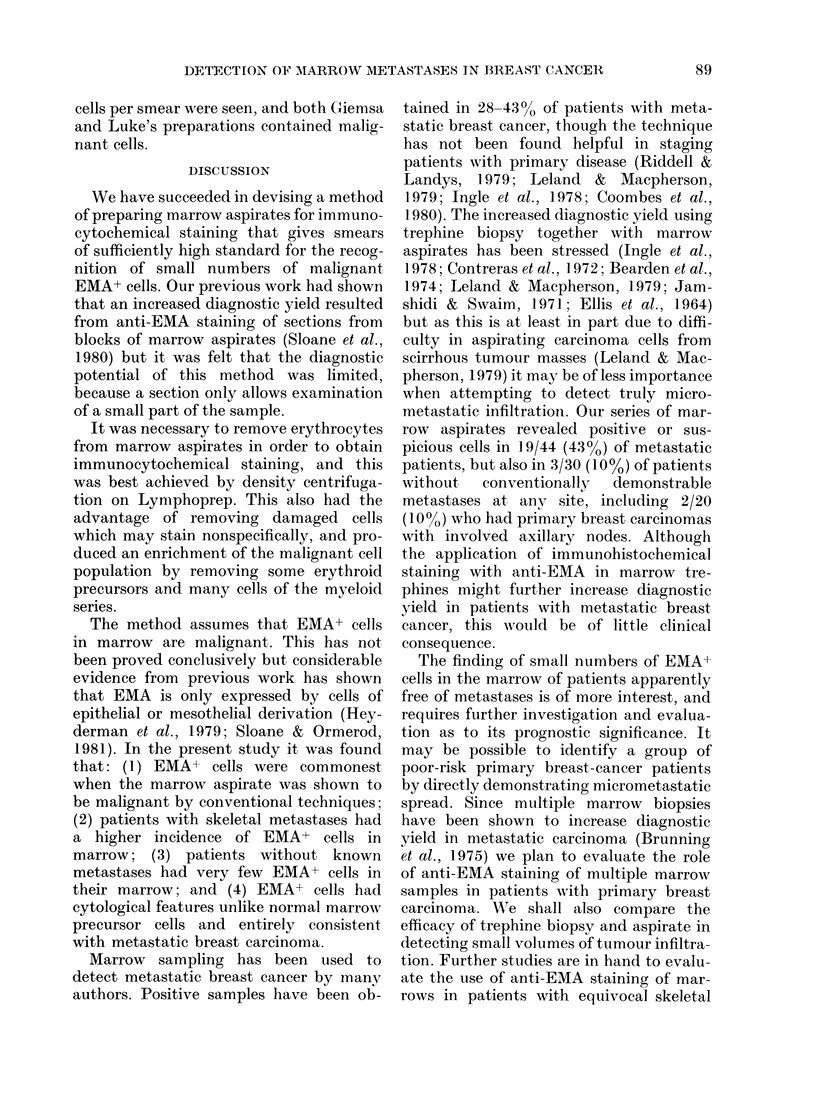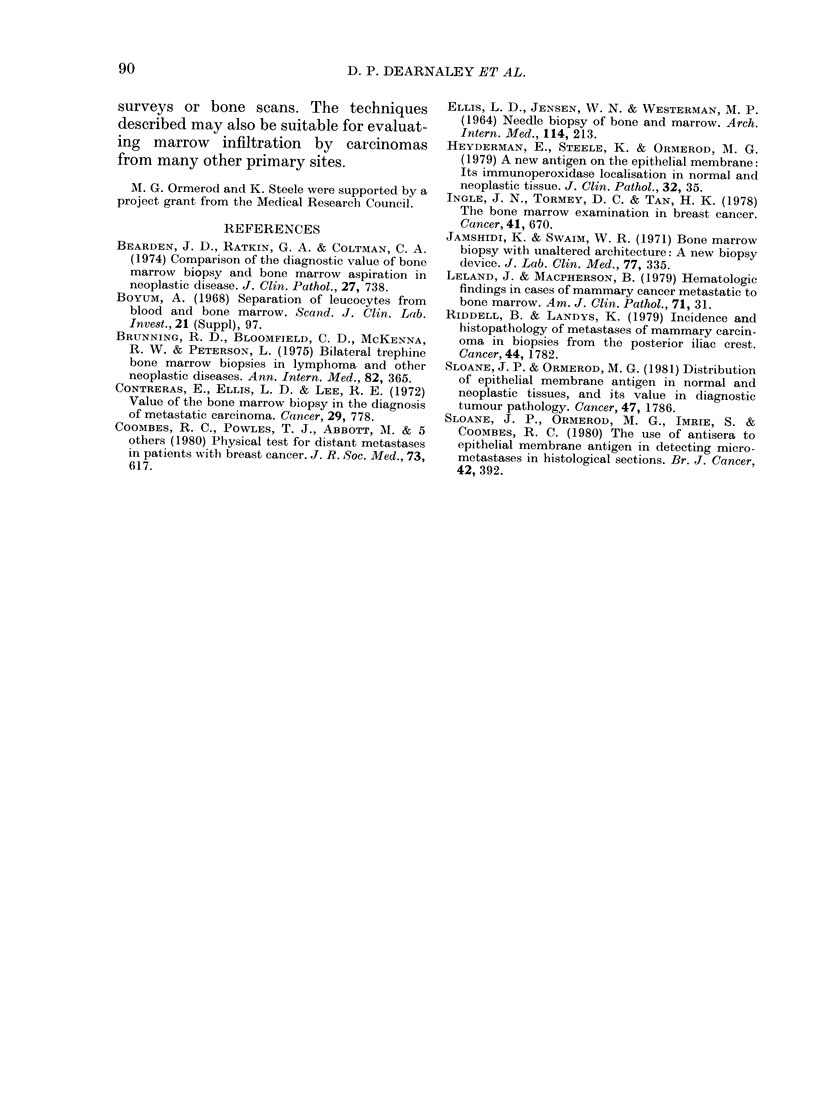# Increased detection of mammary carcinoma cells in marrow smears using antisera to epithelial membrane antigen.

**DOI:** 10.1038/bjc.1981.152

**Published:** 1981-07

**Authors:** D. P. Dearnaley, J. P. Sloane, M. G. Ormerod, K. Steele, R. C. Coombes, H. M. Clink, T. J. Powles, H. T. Ford, J. C. Gazet, A. M. Neville

## Abstract

**Images:**


*Br. J. Cancer* (1981) **44**, 85

### INCREASED DETECTION OF MAMMARY CARCINOMA CELLS IN MARROW SMEARS USING ANTISERA TO EPITHELIAL MEMBRANE ANTIGEN

D. P. DEARNALEY*, J. P. SLOANE†, M. G. ORMEROD‡, K. STEELE‡, R. C. COOMBES*, H. Mc. D. CLINK,† T. J. POWLES† ‡, H. T. FORD†, J.-C. GAZET† AND A. M. NEVILLE*

*From the *Ludwig Institute for Cancer Research (London Branch), Royal Marsden Hospital; †Royal Marsden Hospital, and ‡Institute of Cancer Research: Royal Cancer Hospital, Haddow Laboratories, all at Sutton, Surrey SM2 5PX*

Received 13 January 1981 Accepted 19 March 1981

**Summary.—We have developed a technique for the immunocytochemical staining of marrow smears using antiserum to epithelial membrane antigen (EMA). This membrane component is confined to, but widely distributed in, epithelial tissues and tumours derived from them, and is strongly expressed by infiltrating breast carcinoma cells. Marrow aspirates from patients with both early and metastatic breast cancer have been examined, and the results of immunocytochemical staining compared with conventional cytology and histology. Staining with antiserum to EMA enabled us to detect small numbers of carcinoma cells, and increased the yield of positive samples. Furthermore, using this technique, we found malignant cells in the marrow of patients with primary breast cancer with no other evidence of metastatic disease. Thus immunocytochemical staining for EMA may be of value in the detection of micrometastases in patients with primary breast carcinoma.**

THE LOCALIZATION of a cell-surface component, which has been called epithelial membrane antigen (EMA), has been described in both normal and neoplastic tissue, using an antiserum raised against human milk-fat-globule membranes (Heyderman *et al.*, 1979; Sloane & Ormerod, 1981). In formalin-fixed paraffin-embedded sections, the antigen is confined to, but widely distributed in, epithelial tissues and tumours derived from them. Primary and metastatic breast carcinomas almost always express the antigen (Sloane & Ormerod, 1981) and single cells in infiltrates have been found to stain especially strongly with antisera to EMA. Normal and neoplastic, haemopoietic, lymphoid, osseous and other connective tissues do not express EMA.

Antiserum to EMA has been used to facilitate detection of micrometastases in histological tissue section. Examination of blocks of marrow aspirates from patients with metastatic breast cancer led to the identification of single malignant cells, which increased the yield of positive samples by 21% (Sloane *et al.*, 1980). When only small numbers of malignant cells are present, taking a section from a tissue block introduces considerable sampling errors. Diagnostic yield might further improve by using the antiserum on marrow smears, when the entire aspirated sample may be examined.

In this paper we describe a method of preparing marrow aspirates in a form suitable for subsequent immunocytochemical staining, and demonstrate the value of antisera to EMA in identifying marrow metastases in patients with breast cancer.

### PATIENTS AND METHODS

*Patients.*—Seventy-four marrow aspirates from 71 patients with breast cancer were examined. At the time of sampling patients fell into 3 categories:

(1) 20 patients with primary cancer without evidence of metastatic spread;

(2) 10 patients without evidence of metastases when undergoing staging investigations during follow-up after primary treatment; and

(3) 44 patients with metastatic breast cancer, including 24 with bone metastases.

All these patients were staged as previously described (Coombes *et al.*, 1980). Bone metastases were diagnosed after radiological skeletal survey and isotope bone scan using $^{99m}$technetium diphosphonate.

*Marrow aspirates.*—All aspirates were obtained from the posterior iliac crest. Air-dried smears were made for routine cytological examination and subsequently stained automatically with May-Grunwald Giemsa stain. An aliquot of the remaining sample was processed for immunocytochemistry, and the rest of the aspirate was fixed, embedded, sectioned and stained with haematoxylin and eosin (Luke's preparation) as previously described (Sloane *et al.*, 1980).

*Preparation of marrow aspirates for immunocytochemical staining.*—It was found that routinely prepared marrow smears failed to stain with anti-EMA, despite the presence of obvious clumps of carcinoma cells. This was thought to be due to a failure of primary and/or secondary antiserum to diffuse through layers of dried marrow aspirate. If red cells were removed and thin smears prepared, strong staining of carcinoma cells was apparent. Removal of red cells could be effected either by lysis with ammonium chloride or by density separation using a solution of Ficoll and sodium metrizoate (Boyum, 1968). The latter method gave more reliable results, and was chosen for this study. A density of 1·077 was found suitable (Lymphoprep; Nyegaard); most malignant cells were retained at the interface, together with many cells of the lymphoid series, megakaryocytes, and variable numbers of erythroid and myeloid precursors. The spun pellet contained erythrocytes, normoblasts and some cells of the myeloid series, together with damaged cells. Adequate washing of cells before smearing was necessary to prevent background staining of non-cellular material.

About 0·3 ml of marrow aspirate was taken directly into 0·5 ml Hepes-buffered TC119 medium with 100 u of sterile heparin. This cell suspension was layered on to 10 ml of Lymphoprep in a 30ml Sterilin Universal container and spun at 400 *g* for 20 min. The cell layer at the interface was aspirated, washed twice with TC119 and azide-free, phosphate-buffered saline (PBS). The washed pellet was gently pipetted to disaggregate clumps of loosely cohesive cells. Thin smears were made and fixed immediately in 100% ethanol for 2 h. Smears were stored at room temperature in the dark before staining; deterioration of antigen was not observed over a 2-week period, but storage in the dark at −20°C is recommended for longer intervals.

*Immunohistochemical stain.*—Ethanol-fixed smears were first treated with 2·28% periodic acid in distilled water for 10 min, followed by 0·02% fresh sodium borohydride for a further 2 min. These reagents have been found empirically to abolish nonspecific staining of some haemopoietic cells by the alkaline phosphatase-conjugated second antibody. The smears were next immersed in 20% acetic acid for 10 min to block endogenous alkaline phosphatase present in osteoblasts and leucocytes, and then washed in PBS. Staining was performed using rabbit anti-EMA at a suitable dilution for 90 min, followed by sheep anti-rabbit alkaline-phosphatase-conjugated second antibody for a further 90 min. Sections were washed in PBS between these two steps. Dilutions of both first and second antibodies were made in 1:20 non-immune goat serum and were determined by staining tissue sections of breast carcinomas and smears of breast-carcinoma cell lines. Alkaline phosphatase was visualized using naphthol AS:BI and Brentamine Fast Red TR as substrates for 1 h, after which sections were washed in distilled water and counterstained with Mayer's haemalum for 30 min. To confirm antibody specificity human breast-carcinoma sections were incubated with anti-serum which had been absorbed with a preparation of EMA (Sloane *et al.*, 1980; Sloane & Ormerod, 1981); staining activity was completely abolished.

Alkaline-phosphatase conjugates were preferred to those using horseradish peroxidase as they appear to give more dependable results, and because the technique for blocking endogenous enzyme has less damaging effects on cytological detail. It was also found to be visually easier to screen smears with a red rather than a brown reaction product.

(1)

(2)

(3)

FIG. 1.—Indirect immuno-alkaline phosphatase stain for EMA on marrow smear, showing a clump of breast-carcinoma cells with strong membrane accentuation. ×600.

FIG. 2.—Clump of cells showing granular pattern of positivity to EMA. Membrane accentuation is not apparent and the aspirate was classified as EMA-suspicious (±). Luke's preparation contained a single tumour clump. ×600.

FIG. 3.—Single EMA$^{+}$ cell in marrow smear. Conventional methods showed no evidence of marrow infiltration. ×600.

Smears stained with anti-EMA were read (D.D., J.P.S.) without knowledge of the patient's clinical state or of the results of the Giemsa or Luke's preparations.

### RESULTS

On the smears stained with anti-EMA, clumps of cells, which on morphological grounds could be identified as malignant, gave a strongly positive reaction. The cytoplasm was heavily stained, with membrane accentuation (Fig. 1). Single cells showing the same characteristics of staining and morphology were also found (Fig. 3). These aspirates were recorded as $EMA^+$. On some smears, there were cells or clumps of cells in which the morphology or staining was obscured by overlying cells, or the membrane accentuation was absent. An example is shown in Fig. 2, where a clump of cells shows granular staining without membrane accentuation; in this case the Luke's preparation showed a single small clump of carcinoma. In the absence of other $EMA^+$ cells, such aspirates were recorded as suspicious.

In total, $EMA^+$ cells were identified in 15 aspirates, and suspicious cells or clumps (±) were present in a further 6 (Tables I & II). Malignant cells were seen in 8 aspirates by Giemsa or Luke's preparation. Samples from all but 2 of these patients contained $EMA^+$ cells; of these, one had cells categorized as suspicious, and the other sample was very small (Table III). The presence and distribution of metastases in patients with positive marrow samples is shown in Table II. Most patients with marrow metastases (whether demonstrated conventionally or by anti-EMA staining) had bone deposits revealed by skeletal survey or bone scan. Six patients had $EMA^+$ cells in the marrow, without demonstrable bone metastases, and 2 of these were without metastases at any site, including 1 patient with primary breast cancer involving axillary lymph nodes. Many smears stained with anti-EMA contained a very small number of positive cells. These cells were individually counted and their total number compared with the detection of infiltration by conventional techniques (Table III). Aspirates containing $>100$ $EMA^+$ cells per smear were all detected by conventional morphological methods, whereas only 2/11 aspirates containing $<100$ $EMA^+$ cells were detected from Luke's or Giemsa preparations. The 2 patients without evidence of disseminated cancer had $<5$ $EMA^+$ cells per smear. In one patient with an EMA smear containing about 20 positive cells, conventional methods were negative. On repeat aspiration 4 months later, $>100$ positive

TABLE I.—*Comparison of anti-EMA, Giemsa, and Luke's Preparations in detecting marrow infiltration in* 74 *biopsy samples*

| | EMA | Giemsa | Luke's* |
|---|---|---|---|
| + | 15 | 6 | 5 |
| ± | 6 | 3 | 0 |
| – | 53 | 65 | 66 |

* Inadequate samples in 3 patients.

TABLE II.—*Clinical state of breast-cancer patients and detection of marrow infiltration using either antiserum to EMA or conventional methods*

| | Total No. | EMA | | Giemsa/ Luke's | |
|---|---|---|---|---|---|
| | | + | ± | + | ± |
| Primary, no metastases* | 20 | 1 | 1 | 0 | 0 |
| Post-primary, no metastases* | 10 | 1 | 0 | 0 | 0 |
| Bone metastases | 24 | 9 | 3 | 6 | 2 |
| Metastases (not bone) | 20 | 4 | 2 | 2 | 1 |
| Total | 74 | 15 | 6 | 8 | 3 |

* For screening techniques, see Patients and Methods.

TABLE III.—*Relationship of number of* $EMA^+$ *cells to the detection of marrow infiltration by Giemsa or Luke's preparations*

| $EMA^+$ cells/ smear | No. | $Giemsa^+$ | Luke's* |
|---|---|---|---|
| > 100 | 5 | 5 | 4/4 |
| 5–100 | 3 | 0* | 0 |
| 1–5 | 7 | 0* | 0 |
| 0 | 1 | 1 | 0 |
| Suspicious (< 5) | 6 | 0* | 1 |

* One suspicious sample.

cells per smear were seen, and both Giemsa and Luke's preparations contained malignant cells.

### DISCUSSION

We have succeeded in devising a method of preparing marrow aspirates for immunocytochemical staining that gives smears of sufficiently high standard for the recognition of small numbers of malignant $EMA^+$ cells. Our previous work had shown that an increased diagnostic yield resulted from anti-EMA staining of sections from blocks of marrow aspirates (Sloane *et al.*, 1980) but it was felt that the diagnostic potential of this method was limited, because a section only allows examination of a small part of the sample.

It was necessary to remove erythrocytes from marrow aspirates in order to obtain immunocytochemical staining, and this was best achieved by density centrifugation on Lymphoprep. This also had the advantage of removing damaged cells which may stain nonspecifically, and produced an enrichment of the malignant cell population by removing some erythroid precursors and many cells of the myeloid series.

The method assumes that $EMA^+$ cells in marrow are malignant. This has not been proved conclusively but considerable evidence from previous work has shown that EMA is only expressed by cells of epithelial or mesothelial derivation (Heyderman *et al.*, 1979; Sloane & Ormerod, 1981). In the present study it was found that: (1) $EMA^+$ cells were commonest when the marrow aspirate was shown to be malignant by conventional techniques; (2) patients with skeletal metastases had a higher incidence of $EMA^+$ cells in marrow; (3) patients without known metastases had very few $EMA^+$ cells in their marrow; and (4) $EMA^+$ cells had cytological features unlike normal marrow precursor cells and entirely consistent with metastatic breast carcinoma.

Marrow sampling has been used to detect metastatic breast cancer by many authors. Positive samples have been obtained in 28–43% of patients with metastatic breast cancer, though the technique has not been found helpful in staging patients with primary disease (Riddell & Landys, 1979; Leland & Macpherson, 1979; Ingle *et al.*, 1978; Coombes *et al.*, 1980). The increased diagnostic yield using trephine biopsy together with marrow aspirates has been stressed (Ingle *et al.*, 1978; Contreras *et al.*, 1972; Bearden *et al.*, 1974; Leland & Macpherson, 1979; Jamshidi & Swaim, 1971; Ellis *et al.*, 1964) but as this is at least in part due to difficulty in aspirating carcinoma cells from scirrhous tumour masses (Leland & Macpherson, 1979) it may be of less importance when attempting to detect truly micrometastatic infiltration. Our series of marrow aspirates revealed positive or suspicious cells in 19/44 (43%) of metastatic patients, but also in 3/30 (10%) of patients without conventionally demonstrable metastases at any site, including 2/20 (10%) who had primary breast carcinomas with involved axillary nodes. Although the application of immunohistochemical staining with anti-EMA in marrow trephines might further increase diagnostic yield in patients with metastatic breast cancer, this would be of little clinical consequence.

The finding of small numbers of $EMA^+$ cells in the marrow of patients apparently free of metastases is of more interest, and requires further investigation and evaluation as to its prognostic significance. It may be possible to identify a group of poor-risk primary breast-cancer patients by directly demonstrating micrometastatic spread. Since multiple marrow biopsies have been shown to increase diagnostic yield in metastatic carcinoma (Brunning *et al.*, 1975) we plan to evaluate the role of anti-EMA staining of multiple marrow samples in patients with primary breast carcinoma. We shall also compare the efficacy of trephine biopsy and aspirate in detecting small volumes of tumour infiltration. Further studies are in hand to evaluate the use of anti-EMA staining of marrows in patients with equivocal skeletal surveys or bone scans. The techniques described may also be suitable for evaluating marrow infiltration by carcinomas from many other primary sites.

M. G. Ormerod and K. Steele were supported by a project grant from the Medical Research Council.

REFERENCES

BEARDEN, J. D., RATKIN, G. A. & COLTMAN, C. A. (1974) Comparison of the diagnostic value of bone marrow biopsy and bone marrow aspiration in neoplastic disease. *J. Clin. Pathol.*, **27**, 738.

BOYUM, A. (1968) Separation of leucocytes from blood and bone marrow. *Scand. J. Clin. Lab. Invest.*, **21** (Suppl), 97.

BRUNNING, R. D., BLOOMFIELD, C. D., MCKENNA, R. W. & PETERSON, L. (1975) Bilateral trephine bone marrow biopsies in lymphoma and other neoplastic diseases. *Ann. Intern. Med.*, **82**, 365.

CONTRERAS, E., ELLIS, L. D. & LEE, R. E. (1972) Value of the bone marrow biopsy in the diagnosis of metastatic carcinoma. *Cancer*, **29**, 778.

COOMBES, R. C., POWLES, T. J., ABBOTT, M. & 5 others (1980) Physical test for distant metastases in patients with breast cancer. *J. R. Soc. Med.*, **73**, 617.

ELLIS, L. D., JENSEN, W. N. & WESTERMAN, M. P. (1964) Needle biopsy of bone and marrow. *Arch. Intern. Med.*, **114**, 213.

HEYDERMAN, E., STEELE, K. & ORMEROD, M. G. (1979) A new antigen on the epithelial membrane: Its immunoperoxidase localisation in normal and neoplastic tissue. *J. Clin. Pathol.*, **32**, 35.

INGLE, J. N., TORMEY, D. C. & TAN, H. K. (1978) The bone marrow examination in breast cancer. *Cancer*, **41**, 670.

JAMSHIDI, K. & SWAIM, W. R. (1971) Bone marrow biopsy with unaltered architecture: A new biopsy device. *J. Lab. Clin. Med.*, **77**, 335.

LELAND, J. & MACPHERSON, B. (1979) Hematologic findings in cases of mammary cancer metastatic to bone marrow. *Am. J. Clin. Pathol.*, **71**, 31.

RIDDELL, B. & LANDYS, K. (1979) Incidence and histopathology of metastases of mammary carcinoma in biopsies from the posterior iliac crest. *Cancer*, **44**, 1782.

SLOANE, J. P. & ORMEROD, M. G. (1981) Distribution of epithelial membrane antigen in normal and neoplastic tissues, and its value in diagnostic tumour pathology. *Cancer*, **47**, 1786.

SLOANE, J. P., ORMEROD, M. G., IMRIE, S. & COOMBES, R. C. (1980) The use of antisera to epithelial membrane antigen in detecting micrometastases in histological sections. *Br. J. Cancer*, **42**, 392.